# Protein quality control: from molecular mechanisms to therapeutic intervention—EMBO workshop, May 21–26 2023, Srebreno, Croatia

**DOI:** 10.1007/s12192-023-01383-4

**Published:** 2023-09-20

**Authors:** Christian Münch, Janine Kirstein

**Affiliations:** 1 Institute of Biochemistry II, Medical Faculty, Goethe University Frankfurt, Frankfurt am Main, Germany; 2 Friedrich-Schiller-Universität Jena, Jena, Germany; 3 Leibniz-Institute on Aging/Fritz-Lipmann Institute, Jena, Germany

**Keywords:** Proteostasis, Protein aggregation, Condensation, Molecular chaperones, Proteases

## Abstract

Protein quality control pathways ensure a functional proteome and rely on a complex proteostasis network (PN) that is composed of molecular chaperones and proteases. Failures in the PN can lead to a broad spectrum of diseases, including neurodegenerative disorders like Alzheimer’s, Parkinson’s, and a range of motor neuron diseases. The EMBO workshop “Protein quality control: from molecular mechanisms to therapeutic intervention” covered all aspects of protein quality control from underlying molecular mechanisms of chaperones and proteases to stress signaling pathways and medical implications. This report summarizes the workshop and highlights selected presentations.

## Introduction

**Fig. 1:**
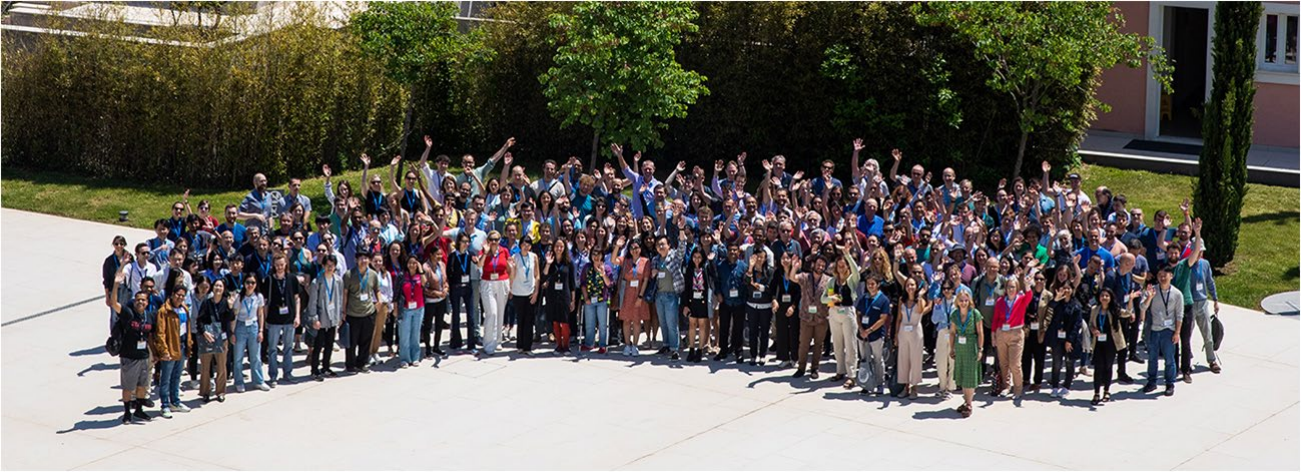
Group picture of all attendees of the EMBO workshop “Protein quality control: from molecular mechanisms to therapeutic intervention”

The EMBO workshop “Protein quality control: from molecular mechanisms to therapeutic intervention” took place in Srebreno at the Adriatic coast of Croatia from May 21st to 26th 2023 and was organized by Eilika Weber-Ban (chair) and Ulrich Hartl (co-chair). Two hundred twenty-four scientists attended the workshop, and the vast majority was from Europe (>70%), followed by North America (12%) and Asia (8%). The workshop was generously sponsored by EMBO and co-sponsored by the Cell Stress Society International (CSSI), ETH Zurich, MPI for Biochemistry, DBIOL, Boehringer Ingelheim, Roche, *Journal of Biological Chemistry*, and NanoTemper. Awards for best oral and poster presentations were sponsored by *FEBS Journal* and *The EMBO Journal*/*EMBO Reports* (Fig. 1).

## Young Scientist Symposium “Protein Quality Control”

This bi-annual EMBO workshop was originally planned for 2021 but had to be postponed due to the COVID-19 pandemic to 2023. To bridge this 2-year gap, the two organizers Eilika Weber-Ban and Ulrich Hartl proposed a virtual Young Scientist Symposium (YSS) “Protein Quality Control” to give early career scientists a chance to present and discuss their work during the pandemic that prohibited any in-person conferences. The absence of meetings hit early career scientists much harder than established PIs as a large part of their PhD or Postdoc training fell into the pandemic with limited lab access and almost no chance for scientific exchange on international conferences.

The YSS was organized by Matthias Block, Tatjana von Rosen from the Weber-Ban lab (ETH Zurich, Switzerland), and Michael Gropp and Max Garhammer from the Hartl lab (MPI Martinsried, Germany). These four PhD students took care of setting up a program, a symposium website, inviting keynote speakers, reviewing abstracts for the selection of presentations, and were also running the actual symposium with support from their PIs and mentors, Eilika Weber-Ban and Ulrich Hartl. This 2-day YSS took place on September 23rd and 24th 2021 and gave 250 PhD students and Postdocs an opportunity to present and discuss their research. Keynote talks by established PIs (Judith Frydman, Claudio Joazeiro, Andreas Martin, Ronald Melki, Reut Shalgi, and Rebecca Taylor) accompanied the scientific program of 18 short and 20 flash talks as well as virtual poster presentations. The YSS received very positive reviews and contributed to the scientific exchange of the whole proteostasis community.

## Keynote lectures

The workshop was opened in the evening of the day of arrival by two keynote lectures. Sheena Radford (University of Leeds) presented data on α-synuclein aggregation. Her lab together with the Brockwell and van Oosten-Hawle labs identified and characterized a short motif in the N-terminal domain of α-synuclein flanking the non-amyloid β component (NAC) domain, P1. P1 is a 7 amino acid long stretch (^36^GVLYVGS^42^) and required for membrane remodeling of the presynaptic α-synuclein. Mutation of Y39A and S42A extends the lag phase of α-synuclein aggregation in vitro and reduces proteotoxicity in vivo in a *Caenorhabditis elegans* model (Doherty et al. 2020; Ulamec et al. 2022). In addition, residue 38 was shown to have a modulatory effect on the amyloid fibril formation of α-synuclein. L38I enhances the kinetics of fibrillization, whereas L38A has no effect and L38M, as found in γ-synuclein, delays the aggregation. NMR studies showed that P1 synergizes with residues within the NAC region and the C-terminal region of α-synuclein to form conformational species capable to initiate amyloid fibrils. Thus, P1 is a peptide stretch that forms weak and transient interactions with other regions within α-synuclein to modulate amyloid formation and may represent a potential target structure for therapeutic intervention.

The second keynote speaker was Bernd Bukau who showed data on selective ribosome profiling. Using single molecule optical tweezer experiments where one ribosome is tethered to one bead and the nascent chain to another, he, in cooperation with Günter Kramer and Sander Tans, established a system to study the sequential binding of trigger factor (TF) and DnaK to nascent chains. In addition, he presented data of ongoing studies on substrate transfer between the ribosome-associated chaperones of the ribosome-associated complex (RAC).

## Molecular chaperone mechanisms, networks, and regulation

The first conference day and first session were opened by Johannes Buchner, who tackled the question how protein substrates are transferred between distinct chaperone systems. It is established that different chaperone systems such as Hsp70 and Hsp90 functionally cooperate with each other. Hsp90 is involved in the later stages of protein folding and maturation and can receive protein substrates from Hsp70 (Boysen et al. 2019; Dahiya et al. 2022; Morán Luengo et al. 2018). Johannes Buchner could show that the co-chaperone NudC (nuclear distribution C) is a transfer factor that can interact with the Hsp70-J-domain protein (JDP)-protein substrate complex. The interaction of NudC with the 70/JDP/substrate complex displaces Hsp70. In a second step, NudC interacts with Hsp90 forming a ternary complex with the substrate-bound JDP and Hsp90 that allows the protein substrate transfer from the JDP to Hsp90 (Biebl et al. 2022).

To identify physico-chemical parameters within Hsp90 substrates, Brian Freeman used a photoactivatable-crosslinking mass spectrometry approach. He could show that Hsp90 interacts with as much as 20% of all proteins in yeast. Hsp90 binds its clients with all three domains and preferentially binds to intrinsically disordered domains (IDRs). The interaction of Hsp90 prevented the transition of IDR-containing proteins into, e.g., stress granules and P-bodies. Hsp90 was also found to associate with the 48S ribosome and thereby regulating translation initiation that—if perturbed—activates the heat shock response (Kolhe et al. 2023).

Ulrich Hartl presented a new GCN1 (general control non-depressible protein 1)-mediated mechanism on how aberrant proteins that can be produced upon readthrough into the 3′ untranslated region (UTR) of the mRNA can be eliminated (Müller et al. 2023). His lab could show that readthrough proteins are cleared by a coupled process involving Bcl-2-associated athanogene 6 (BAG6) and the ribosome-collision-sensing protein GCN1. Readthrough proteins that carry hydrophobic C-terminal extensions are recognized by BAG6 and get ubiquitylated by the E3 ligase RNF126 for the subsequent proteasomal degradation. The readthrough mRNAs are cleared by GCN1 that recognizes ribosomes that, e.g., collide at non-optimal codons in 3′UTRs and then recruits CCR4/NOT for mRNA decay. GCN1 is highly conserved and required for stress signaling and may play an important role in translational surveillance during aging.

The interaction of molecular chaperones and their substrate proteins is often of transient nature and rather weak. Heath Ecroyd showed how single molecular fluorescence resonance energy transfer (smFRET) can be used to study conformational changes during the folding process and to determine chaperone-protein substrate stoichiometries. smFRET was applied to study the interaction between the sHsp alphaB-crystallin (αBc) and the protein substrate chloride intracellular channel 1 (CLIC1). αBc forms large polydisperse assemblies that are difficult to study mechanistically. smFRET overcomes these challenges and enabled the direct analysis of αBc and CLIC1 complexes. αBc inhibits the formation of CLIC1 aggregates in response to, e.g., heat in favor of dynamic polydisperse αBc-CLIC1 complexes (Johnston et al. 2021). A two-step mechanism of sHsp-client formation was proposed where sHsps initially recognize and bind misfolded substrate proteins. These complexes are in a second step recognized by additional free sHsps to form larger sHsp-substrate complexes until an equilibrium of bound and unbound sHsp is reached.

Axel Mogk presented data on the AAA+ protein ClpG, a protein disaggregase with homology to the well-characterized ClpB. Unlike ClpB, ClpG does not cooperate with the DnaK, DnaJ, GrpE system and has a very high basal ATPase and disaggregation activity. ClpG is encoded on a transmissible locus such as a mobile genomic island or plasmid and plays a role as virulence factor and promotes bacterial survival of the pathogens *Klebsiella pneumoniae* and *Pseudomonas aeruginosa*. Compared to ClpB, ClpG possesses a distinct M domain, a N-terminal domain 1 (NTD1), and a C-terminal extension. Axel Mogk could show that the NTD1 is essential for substrate recognition and disaggregation. The ATPase activity of ClpG is inhibited by NTD1 and NTD2. This inhibition is released upon substrate binding by NTD1 and thereby regulates the ATPase and chaperone activity and prevents wasteful ATPase cycles. The disaggregation of protein aggregates occurs by threading the substrate through the pore of the hexameric ClpG protein complex (Katikaridis et al. 2021).

The sHsp clusterin is a secreted, extracellular holdase-type chaperone found to be a risk factor for Alzheimer’s disease accumulating in patient brains and plaques. Patricia Yuste Checa from the Hartl lab presented a clusterin crystal structure and showed in vitro that clusterin prevents Abeta aggregation. The hydrophobic and flexible tail of clusterin is required and sufficient for the suppression of Abeta aggregation. Interestingly, whereas clusterin can prevent the aggregation for some amyloids, it can produce more seeding-competent conditions for other amyloid proteins. Thus, the complex effects of clusterin shown by the Hartl lab in Parkinson’s disease expand to Alzheimer’s disease (Yuste-Checa et al. 2021, 2022).

Judith Frydman reported on a new study on the coordination of nuclear and cytosolic protein quality control. Using a yeast model and soft X-ray tomography, she showed that the deposits for misfolded proteins, INQ and JUNQ, align in proximal positions near nuclear-vacuolar junctions and communicate across compartments via the nuclear pore. Budding of nuclear regions including INQ allows the transfer of INQ into the vacuole for clearance. This process requires the ESCRT machinery in a Vps4-dependent manner (Sontag et al. 2023).

Changes in transcription are a main driver of cellular changes upon proteostasis perturbation (Aprile-Garcia et al. 2019). Ritwick Sawarkar presented new work on stress-induced transcriptional attenuation (SITA). In an aim to create SITA-incompetent mice, SITA components were knocked out, leading to neuroinflammation and neurological disorders. Based on a genome-wide screen for negative elongation factor (NELF) condensate modulators, he identified a new factor termed SITAm-6 that links the transcriptional reduction during SITA with translational effects.

Insight into the molecular mechanism of Hsc70 in regulating the heat shock response came from Matthias Mayer, who showed that the combination of Hsc70, DNAJB1, and ATP is required to remove the heat shock transcription factor Hsf1 from DNA (Kmiecik et al. 2020). Functionally, Hsc70 binds to multiple sites in Hsf1 and thereby monomerizes Hsf1 to attenuate the heat shock response.

Arno Alpi concluded this session and presented the supramolecular assembly of the glucose-induced degradation-deficient (GID)/CTHL E3 ligase, which is driven by Gid7 (Sherpa et al. 2021). He showed that the substrate receptor Gid4 is not required for the complex formation and introduced NMNAT1 as a new interactor.

## Protein quality control at the ribosome

The next session focused on the quickly evolving field of protein quality control at the ribosome, particularly in a co-translational manner. Elke Deuerling presented structures that were obtained in cooperation with Nenad Ban and that show how NAC binds to the ribosome (Jomaa et al. 2022) and is required for the recruitment of the methionine aminopeptidases (METAPs) (Gamerdinger et al. 2023). The flexible NAC tail binds and thereby recruits METAP1 to the ribosome for the removal of methionine from cytosolic proteins. Nascent chains containing an ER signal sequence are bound by the competing signal recognition particle (SRP) and escape the methionine excision.

Irmgard Sinning reported on the ribosome-associated complex (RAC) that forms a functional chaperone triad with the Hsp70 Ssb and the J-domain protein Zuotin. Cryo-EM structures of *Chaetomium thermophilum* RAC bound to the 80S ribosome showed two different conformations that accommodate continuous ribosomal rotation (Kišonaitė et al. 2023). The non-canonical Hsp70 Ssz1 and Zuotin form a rigid unit. The HPD motif of Zuotin is masked by the nucleotide binding domain (NBD) of Ssz1 that thereby prevents premature activation of the Hsp70 Ssb at the ribosome. The elongating nascent chain that emerges from the ribosomal exit tunnel can push away the NDB of Ssz1 to release the HPD motif of Zuotin. The HPD motif of Zuotin can now be bound by Ssb whose ATPase activity is then activated.

To gain insight into co-translational folding in bacterial, David Balchin studied nascent chain interactions with trigger factor (TF), DnaK, and DnaJ. Structural crosslinking mass spectrometry showed that each factor exhibited a binding preference that depends on nascent chain lengths. These data suggest successive interaction of the chaperones with the nascent chain to aid in the folding of the newly synthesized protein.

During oxidative stress, *Saccharomyces cerevisiae* activates a stress response pathway mediated by polysome-interacting La-related proteins Slf1 and Sro9 to adjust protein translation. Priya Srivastava from the Pavitt lab presented photoactivatable ribonucleoside-enhanced crosslinking and immunoprecipitation (PAR-CLIP) experiments to determine Slf1 and Sro9 binding sites on mRNAs in stressed and non-stressed yeast. These experiments revealed that Slf1 supports cellular stress adaptation by stabilizing ribosomes translating antioxidant proteins. Molecularly, Slf1 prevents ribosome frameshifting due to ribosome stalling during stress and thus promotes translation of proteins critical for the stress response (Jennings et al. 2023).

Ribosome-associated quality control (RQC) targets truncated nascent peptides produced from ribosome stalling for degradation. Central components in mammals are the human ribosome- and tRNA-binding protein NEMF that stabilizes the E3 ligase LTN1/Listerin on large ribosomal subunits obstructed with an incomplete nascent chain. These chains are then ubiquitinated by Listerin and subsequently degraded by the proteasome (Filbeck et al. 2022). In addition, NEMF can mediate C-terminal alanine tailing of stalled peptides to create a degron (Thrun et al. 2021). Claudio Joazeiro presented two E3 ligases, Pirh2 and CRL2-KLHDC10, which bind to the alanine tail degrons and mediate their degradation. In addition, he provided structural insight into the binding mode and further established a widely conserved role of alanine tailing in protein quality control (Patil et al. 2023).

Onn Brandman focused on carboxy-terminal alanine and threonine residues (CAT tails), introduced during the RQC process by Rqc2p/NEMF. CAT tails promote the targeting of structured stalled polypeptides by Ltn1 (Sitron and Brandman 2019). He characterized different CAT-tailed proteins to identify guiding features toward their degron propensities. These findings provided novel insight into an understanding of how CAT tails can have beneficial effects during RQC, while being potentially harmful due to the aggregation propensity of CAT-tailed peptides (Sitron et al. 2020).

## Protein homeostasis and quality control in organelles and cellular compartments

Protein quality pathway of the organelles and their integration into cellular stress pathways were the focus on the second conference day. The morning session focused on the endoplasmic reticulum (ER) and the unfolded protein response of the ER (UPR^ER^).

The UPR^ER^ is regulated by different signaling branches. One of them is regulated by IRE1 whose signaling activity is controlled by the ER Hsp70 chaperone BiP. IRE1β is a homolog that is only expressed in mucin-producing cells. David Ron showed that the mucin chaperone and protein disulfide isomerase (PDI) AGR2 is a selective repressor for IRE1β and regulates the UPR^ER^ in a tissue-specific manner. Another PDI, PDIA6, was the focus of a study presented by Anna Leder of the Hiller lab demonstrating that a structural switch regulates condensation of PDIA6 in a Ca^2+^ and redox-dependent manner. PDIA6 is involved in the folding of ER proteins likely within condensates and is a key regulator of the UPR^ER^ via the IRE1α branch.

Cells can enter a reversible cell cycle arrest and limit their proliferation under stress conditions. Using *C. elegans* as model system, Andrew Dillin could show that the UPR^ER^ axis and lysosomal degradation pathways are involved in the regulation of quiescent cells. The connection between the UPR^ER^ and the integrated stress response (ISR) was discussed by Peter Walter who showed that eIF2α phosphorylation affects synaptic plasticity and memory consolidation. The ISR inhibitor ISRIB restored protein synthesis, improved memory formation, and was effective upon traumatic brain injury in murine models. The proposed molecular target of ISRIB is the GTP exchange factor and translation initiation factor 2B (eIF2B) (Anand and Walter 2020).

A new ER stress signaling pathway termed ER-associated RNA silencing (ERAS) was identified and presented by Sotirios Efstathiou of the Hoppe lab (Efstathiou et al. 2022). ERAS is an anti-viral RNA interference pathway that cooperates with ER-associated degradation (ERAD) to preserve ER homeostasis. ERAS is mediated by the conserved Argonaute protein RDE-1/AGO2 leading to RNA silencing and degradation of ER-associated mRNAs to ameliorate the burden of ER proteins.

The afternoon session focused on the mitochondrial protein quality control and how it is embedded into the cellular proteostasis network. The correct targeting of mitochondrial outer membrane proteins requires specific transporters and receptors. Gayathri Muthukumar from the Weissman lab used a CRISPRi screen to identify transporters for α-helical outer membrane proteins. Using this approach, MTCH2 could be identified as novel insertase (Guna et al. 2022). In ongoing studies, they are now screening for cytosolic factors that regulate the correct targeting of mitochondrial outer membrane proteins.

Failures in mitochondrial import can lead to clogging of the import transporters. In contrast to yeast, it was found by the Bragoszewski and Chacinska labs that human cells do not rely on ATPase-driven extraction and subsequent targeting to the proteasome, but use instead mitochondrial factors to clear translocation channel blockage (Krakowczyk et al. 2023). Mitochondrial depolarization activates mitochondrial proteases such as OMA1 to cleave the clogged protein that in turn is released and on the cytosolic side targeted by p97 to the proteasome for complete degradation.

Perturbations of the mitochondrial protein quality control are communicated to the nucleus to mount the mitochondrial unfolded protein response (UPR^mt^). A number or studies have greatly contributed to our understanding of the involved signaling factors (Anderson and Haynes 2020; Berendzen et al. 2016; Labbadia and Morimoto 2015), but much less was known for mammalian cells (Münch 2018). Christian Münch presented data that showed that release of mitochondrial ROS into the cytosol oxidizes the cytosolic J-domain protein, DNAJA1, that in its oxidized form recruits HSP70 to accumulating mitochondrial protein precursors upon import defects during mitochondrial stress (Michaelis et al. 2022). As shown for other stress signaling mechanism, a competition for a limited pool of chaperones then liberates HSP70 from the transcription factor HSF1. The released HSF1 in turn translocated to the nucleus to activate genes of the UPR^mt^ (Sutandy et al. 2023).

## Cellular protein degradation in quality control

To identify specific substrates for the UPS components, Anton Khmelinskii performed a proteomic screen using fluorescent timer proteins in yeast. His focus was on the glucose-induced degradation-deficient (GID) complex, a large multi-subunit E3 ligase that uses different substrate receptors, Gid4/10 responsible for Pro/N-degron substrates and a novel receptor Ylr149c/Gid11. The Khmelinskii lab could refine the substrate specificity of Gid11 and show that these proteins carry an N-degron with an exposed threonine after removal of the starter methionine. Gid11 protein substrates are glycolytic enzymes and regulate the metabolic switch of yeast from utilizing glucose to ethanol as carbon source (Kong et al. 2021).

The ubiquitin code was the topic of the presentation of Michael Rape. He showed that ubiquitin branching increases with stress, is produced in response to distinct signaling cues and can improve the efficiency of protein degradation, and is involved in the organization of large signaling complexes (Kolla et al. 2022). In addition, he presented data on the role of E3 ligases in converging different cellular stress signaling pathways by recognizing bi-functional motifs in stress inducers and stress response mediators.

Andreas Martin presented mechanistic data on substrate engagement of the AAA+ protein Cdc48 (p97) that uses its ATPase activity for protein substrate extraction and unfolding. Using a FRET-based system, he could show that Cdc48 uses the adaptor Ufd1 to bind a ubiquitin moiety on the proximal side of the initiator ubiquitin and thereby directs the initiator ubiquitin toward rapid unfolding by Npl4 and engagement by Cdc48. Ubiquitin proteins on the distal side increase substrate affinity and facilitate unfolding. Notably, Ufd1/Npl4 hold onto the ubiquitinated substrate even after engagement of Cdc48, presumably to prevent premature substrate escape (Williams et al. 2023). Cdc48 (p97) can also disassemble protein substrates in a ubiquitin-independent manner by using p37 as adaptor protein. Johannes van den Boom of the Meyer lab presented structural data of p97 and the substrate protein phosphatase-1 (PP1) with its partners SDS22 and inhibitor-3 (I3). The obtained structural data suggest a hold-and-extract mechanism for p97-mediated disassembly (van den Boom et al. 2023).

Prokaryotic proteases such as ClpP can be used as target for the development of new antibiotics. Gabrielle Taylor of the Weber-Ban lab presented structural and biochemical data of ClpCP of the pathogenic bacterium *Mycobacterium tuberculosis* and cyclic heptapeptide cyclomarin A (CymA) that exhibits a strong toxicity toward *M. tuberculosis*. Her data shed light onto the mechanism. A co-crystal structure revealed that CymA binds to the N-terminus of ClpC1 and facilitates assembly into an active conformation. Hence, CymA exerts its toxicity by potentiating ClpC1 (Taylor et al. 2022). CymA can also bind to the homologous ClpC2, leading to a competition of both ClpC proteins for CymA. In vivo, CymA treatment leads to an upregulation of ClpC2 to counteract the dysregulation of the ClpCP-dependent degradation by CymA (Taylor et al. 2023).

Proteolysis targeting chimeras (PROTACs) are heterobifunctional compounds containing ligands for a target protein and an E3 ligase, which have shown clinical potential. Tim Clausen presented PROTACs for bacterial proteins of interest, targeting these to ClpC (Morreale et al. 2022). Targeting ClpC1 causes its degradation, along with extensive other effects including induction of the stress-response factors ClpC2 and ClpC3.

The 26S proteasome has been considered as the main proteasomal degradation route. However, proteins can also be cleared by the uncapped 20S proteasome. The identity and selection of substrates for the uncapped 20S proteasome remains unclear (Deshmukh et al. 2023). Michal Sharon presented a modified version of limited proteolysis proteomics method termed PIP (proteasomal induced proteolysis) that allows to monitor 20S substrates. Oxidative stress leads to enhanced 20S activity. She could show that typical protein substrates of the 20S are oxidized and mutated proteins as well as those with intrinsically disordered regions (IDR).

Sara Roas of the Hegde lab presented data on the quality of protein complex assembly, specifically on how orphaned complex subunits are degraded. Focusing on components of the proteasome and the cytosolic chaperonin CCT, she showed an involvement of the E3 ligases HERC1 and HERC2 in the degradation of unassembled CCT subunits (Yagita et al. 2023; Zavodszky et al. 2021).

Proteasome activator subunit 4 (PSME4) is a marker of non-responsiveness to immunotherapy in different cancers. Yifat Merbl reported that increased PSME4 levels in non-small-cell lung carcinoma (NSCLC) alter proteasome complex composition and increase caspase-type digestion over tryptic-like digestion. Changes in PSME4 levels modulate peptide presentation on MHC-I and could explain the observed T cell-mediated immune suppression in lung cancer models (Javitt et al. 2023).

## Mechanisms of phase separation, aggregation, and their pathology

Proteotoxic conditions such as heat shock can promote the assembly of translation factors into condensates. Simon Alberti presented data how such a translation factor and DEAD-box protein, Ded1, senses heat stress. Ded1 undergoes heat-induced structural arrangements that favor condensate formation that are regulated by an interplay between a structured domain and intrinsically disordered regions (Jegers et al. 2022).

The disassembly of stress granules is regulated by molecular chaperones. Hyun Kate Lee presented data on the role of HSP70, different J-domain proteins, and HSP110 in the disassembly of condensates formed by wildtype and mutant FUS as well as G3BP1.

Stress granule (SG) dynamics are also regulated by SUMO-targeted ubiquitin ligase (StUbL) pathway that is part of the nuclear proteostasis network. The ubiquitin-like SUMO system is hence involved in a crosstalk between the nuclear and cytosolic stress pathways. Stefan Müller showed that StUbL contributes to the proteotoxic stress resilience by regulating SG dynamics of, e.g., FUS. In addition, a compromised SUMO-RNF4 axis delays SG disassembly upon relieve of stress conditions (Keiten-Schmitz et al. 2020).

The involvement of small heat shock proteins in the condensation and aggregation of proteins is established. Their substrate specificity and interaction with other biological molecules that affect protein aggregation such as lipids is far less understood. Serena Carra presented data of her lab and the cooperation partner Michele Vendruscolo on the role of sHsps on the aggregation of the presynaptic protein α-synuclein that can be modulated by the presence of lipids and the interaction between sHsps and lipids.

Reut Shalgi screened the expression of chaperones in response to the expression of either FUS or HTTpolyQ and identified naturally occurring isoforms of two J-domain proteins, DNAJB12 and DNAJB14 that form a complex as full-length protein and ameliorated FUS aggregation in an Hsp70-dependent manner. Their shorter isoforms lost the ability to form a complex and did not counteract FUS aggregation anymore. In contrast, the full-length DNAJB12 aggravated and the short isoform of DNAJB12 protected against HTTpolyQ aggregation (Rozales et al. 2022). These data show that the contribution of different isoforms of JDPs adds another layer to the complexity of chaperone networks that show different substrate specificity, mode of action and may also differ in their cooperation with partner chaperones.

## Proteostasis dysfunction in aging and disease

Using a TDP43 *C. elegans* neurodegenerative disease model, Ellen Nollen could show that an impaired release of the neurotransmitters γ-aminobutyric acid (GABA) and acetylcholine (ACh) was the primary defect in this disease model. TDP43 exhibited loss of GABA synapses and silenced ACh neurons. Using an optogenetic approach, her lab could show that a stimulation of repressed ACh neurons could restore neurotransmission. She could further show that a simultaneous activation of GABA and ACh synergistically activated neuronal activity (Koopman et al. 2021). These observations suggest that neuronal circuits should be targeted for therapeutic interventions of neurodegenerative diseases.

Harm Kampinga presented data on the non-canonical J-domain protein DNAJB6 that co-localizes with intrinsically disordered proteins, e.g., FG-nucleoporins (Kuiper et al. 2022) and polyQ condensates. The co-condensation of DNAJB6 with polyQ increased the percentage of cells with mobile condensates and could prevent their transition to solid states (De Mattos et al. 2022). In addition, he showed data on the fragmentation of large protein aggregates by Hsp70 and DNJB6 with a subsequent engulfment of the released fragments into autophagosome and hence further expanding the functional repertoire of DNAJB6. In contrast to DNAJB6, DNAJB1 relies on Hsp70 for its chaperone activities. DNAJB1 is a generalist and has been shown to interact with numerous different protein substrates and folding states. Janine Kirstein presented her recent data on the identification of a substrate-specific binding interface. DNAJB1 binds with its C-terminal hinge region to the proline rich domain of Huntingtin (HTT). Mutating a single aa (H244) in this C-terminal site completely abrogated the ability of DNAJB1 to suppress HTT aggregation together with Hsp70 and Apg2 (Ayala Mariscal et al. 2022).

New insights on the age-associated decline of proteostasis were presented by John Labbadia who showed that the depletion of the outer mitochondrial membrane protein MTCH-1 reduced the aggregation and toxicity of polyQ and Abeta in *C. elegans* (Aman et al. 2022). Interestingly, although MTCH-1 maintains proteostasis capacity with age in an HSF-1-dependent manner, the heat shock response is not required for this process. A model for MTCH-1 was introduced in which decreased MTCH-1 levels enhance HSF-1 activity via inhibition of Hsp90.

Anne Bertolotti presented her efforts over the last years on phosphatase inhibitors that activate the ISR (Das et al. 2015; Krzyzosiak et al. 2018; Tsaytler et al. 2011) and showed similarities and differences to data of Peter Walter with ISRIB (Sidrauski et al. 2013, 2015; Tsai et al. 2018; Zhu et al. 2019), an ISR inhibitor that prevents the downstream effects of eIF2a phosphorylation. She presented a theoretical framework for how ISR is likely at the center of several diseases. Activation and/or inhibition may positively affect cellular and organismal fitness, likely depending on the specific disease conditions. Giving examples of the beneficial effects of raphin 1, Anne Bertolotti showed data from a HD mouse model treated with raphin 1 that led to a rescue of cognitive deficits as well as improved fibroblast to striatal neuron differentiation.

Konstanze Winklhofer reported data on the PQC events downstream of the linear ubiquitin chain assembly complex (LUBAC). Upon protein aggregation, NEMO is recruited to protein aggregates by binding to linear ubiquitin chains produced by HOIP, the catalytic LUBAC component (Wu et al. 2022). These processes amplify linear ubiquitination of protein aggregates and NEMO recruitment that binds to p62 and thereby promotes autophagic clearance of the aggregates (Furthmann et al. 2023). This can explain some of the earlier data by the Winklhofer group that proposed a role of NEMO in controlling protein phase separation (Goel et al. 2023).

The meeting was closed by the final keynote lecture given by Rick Morimoto. He started his talk by reminding the community of the publicly available annotated human proteostasis network (The Proteostasis Consortium 2022, 2023). He then presented data on an analysis of the capacity of the proteostasis network to maintain a functional proteome. He used the nematode *C. elegans* and examined the dynamics of the global proteome conformational stability in animals challenged by the expression of either a temperature-sensitive (ts) mutant (myosin G387R mutant) or by polyQ proteins. Both animal cohorts were assessed in an aging context. Using quantitative proteomics with the Gygi and Finley labs, he compared the networks of metastable proteins and identified only a small number of common proteins. However, more than one third of the entire proteome undergoes conformational changes in early adulthood. The expression of the polyQ proteins accelerates the age-associated destabilizing effect on the proteome (Sui et al. 2022). Aging is the biggest risk factor for neurodegenerative diseases and as shown by data of Rick Morimoto’s lab, the dominating determinant of proteome stability.

We thank Eilika Weber-Ban and Ulrich Hartl for the organization of a very successful meeting and are looking forward to the next EMBO conference on “Protein Quality Control” in 2 years that will be organized by Ulrich Hartl and Serena Carra who was elected as co-organizer.

## References

[CR1] Aman Y, Erinjeri AP, Tataridas-Pallas N, Williams R, Wellman R, Chapman H, Labbadia J (2022). Loss of MTCH-1 suppresses age-related proteostasis collapse through the inhibition of programmed cell death factors. Cell Rep.

[CR2] Anand AA, Walter P (2020). Structural insights into ISRIB, a memory-enhancing inhibitor of the integrated stress response. FEBS J.

[CR3] Anderson NS, Haynes CM (2020). Folding the mitochondrial UPR into the integrated stress response. Trends Cell Biol.

[CR4] Aprile-Garcia F, Tomar P, Hummel B, Khavaran A, Sawarkar R (2019). Nascent-protein ubiquitination is required for heat shock-induced gene downregulation in human cells. Nat Struct Mol Biol.

[CR5] Ayala Mariscal SM, Pigazzini ML, Richter Y, Özel M, Grothaus IL, Protze J, Ziege K, Kulke M, ElBediwi M, Vermaas JV, Colombi Ciacchi L, Köppen S, Liu F, Kirstein J (2022). Identification of a HTT-specific binding motif in DNAJB1 essential for suppression and disaggregation of HTT. Nat Commun.

[CR6] Berendzen KM, Durieux J, Shao L-W, Tian Y, Kim H-E, Wolff S, Liu Y, Dillin A (2016). Neuroendocrine coordination of mitochondrial stress signaling and proteostasis. Cell.

[CR7] Biebl MM, Delhommel F, Faust O, Zak KM, Agam G, Guo X, Mühlhofer M, Dahiya V, Hillebrand D, Popowicz GM, Kampmann M, Lamb DC, Rosenzweig R, Sattler M, Buchner J (2022). NudC guides client transfer between the Hsp40/70 and Hsp90 chaperone systems. Mol Cell.

[CR8] Boysen M, Kityk R, Mayer MP (2019). Hsp70- and Hsp90-mediated regulation of the conformation of p53 DNA binding domain and p53 cancer variants. Mol Cell.

[CR9] Dahiya V, Rutz DA, Moessmer P, Mühlhofer M, Lawatscheck J, Rief M, Buchner J (2022). The switch from client holding to folding in the Hsp70/Hsp90 chaperone machineries is regulated by a direct interplay between co-chaperones. Mol Cell.

[CR10] Das I, Krzyzosiak A, Schneider K, Wrabetz L, D’Antonio M, Barry N, Sigurdardottir A, Bertolotti A (2015). Preventing proteostasis diseases by selective inhibition of a phosphatase regulatory subunit. Science.

[CR11] De Mattos EP, Musskopf MK, Bergink S, Kampinga HH (2022) *In vivo* suppression of polyglutamine aggregation via co-condensation of the molecular chaperone DNAJB6 (preprint). Cell Biol. 10.1101/2022.08.23.504914

[CR12] Deshmukh FK, Ben-Nissan G, Olshina MA, Füzesi-Levi MG, Polkinghorn C, Arkind G, Leushkin Y, Fainer I, Fleishman SJ, Tawfik D, Sharon M (2023). Allosteric regulation of the 20S proteasome by the catalytic core regulators (CCRs) family. Nat Commun.

[CR13] Doherty CPA, Ulamec SM, Maya-Martinez R, Good SC, Makepeace J, Khan GN, van Oosten-Hawle P, Radford SE, Brockwell DJ (2020). A short motif in the N-terminal region of α-synuclein is critical for both aggregation and function. Nat Struct Mol Biol.

[CR14] Efstathiou S, Ottens F, Schütter L-S, Ravanelli S, Charmpilas N, Gutschmidt A, Le Pen J, Gehring NH, Miska EA, Bouças J, Hoppe T (2022). ER-associated RNA silencing promotes ER quality control. Nat Cell Biol.

[CR15] Filbeck S, Cerullo F, Pfeffer S, Joazeiro CAP (2022). Ribosome-associated quality-control mechanisms from bacteria to humans. Mol Cell.

[CR16] Furthmann N, Angersbach L, Bader V, Blusch A, Goel S, Sánchez-Vicente A, Krause LJ, Grover P, Trinkaus VA, Van Well EM, Jaugstetter M, Tschulik K, Damgaard RB, Saft C, Ellrichmann G, Gold R, Koch A, Englert B, Glatzel M et al (2023) NEMO reshapes the protein aggregate interface and promotes aggrephagy by co-condensation with p62 (preprint). Cell Biol. 10.1101/2023.06.05.543428

[CR17] Gamerdinger M, Jia M, Schloemer R, Rabl L, Jaskolowski M, Khakzar KM, Ulusoy Z, Wallisch A, Jomaa A, Hunaeus G, Scaiola A, Diederichs K, Ban N, Deuerling E (2023). NAC controls cotranslational N-terminal methionine excision in eukaryotes. Science.

[CR18] Goel S, Oliva R, Jeganathan S, Bader V, Krause LJ, Kriegler S, Stender ID, Christine CW, Nakamura K, Hoffmann J-E, Winter R, Tatzelt J, Winklhofer KF (2023). Linear ubiquitination induces NEMO phase separation to activate NF-κB signaling. Life Sci Alliance.

[CR19] Guna A, Stevens TA, Inglis AJ, Replogle JM, Esantsi TK, Muthukumar G, Shaffer KCL, Wang ML, Pogson AN, Jones JJ, Lomenick B, Chou T-F, Weissman JS, Voorhees RM (2022). MTCH2 is a mitochondrial outer membrane protein insertase. Science.

[CR20] Javitt A, Shmueli MD, Kramer MP, Kolodziejczyk AA, Cohen IJ, Radomir L, Sheban D, Kamer I, Litchfield K, Bab-Dinitz E, Zadok O, Neiens V, Ulman A, Wolf-Levy H, Eisenberg-Lerner A, Kacen A, Alon M, Rêgo AT, Stacher-Priehse E, Lindner M, Koch I, Bar J, Swanton C, Samuels Y, Levin Y, da Fonseca PCA, Elinav E, Friedman N, Meiners S, Merbl Y (2023). The proteasome regulator PSME4 modulates proteasome activity and antigen diversity to abrogate antitumor immunity in NSCLC. Nat Can.

[CR21] Jegers C, Franzmann TM, Hübner J, Schneider J, Landerer C, Wittmann S, Toth-Petroczy A, Sprangers R, Hyman AA, Alberti S (2022) A conserved and tunable mechanism for the temperature-controlled condensation of the translation factor Ded1p (preprint). Biochemistry. 10.1101/2022.10.11.511767

[CR22] Jennings MD, Srivastava P, Kershaw CJ, Talavera D, Grant CM, Pavitt GD (2023). Interaction of the La-related protein Slf1 with colliding ribosomes maintains translation of oxidative-stress responsive mRNAs. Nucleic Acids Res.

[CR23] Johnston CL, Marzano NR, Paudel BP, Wright G, Benesch JLP, van Oijen AM, Ecroyd H (2021). Single-molecule fluorescence-based approach reveals novel mechanistic insights into human small heat shock protein chaperone function. J Biol Chem.

[CR24] Jomaa A, Gamerdinger M, Hsieh H-H, Wallisch A, Chandrasekaran V, Ulusoy Z, Scaiola A, Hegde RS, Shan S-O, Ban N, Deuerling E (2022). Mechanism of signal sequence handover from NAC to SRP on ribosomes during ER-protein targeting. Science.

[CR25] Katikaridis P, Römling U, Mogk A (2021). Basic mechanism of the autonomous ClpG disaggregase. J Biol Chem.

[CR26] Keiten-Schmitz J, Wagner K, Piller T, Kaulich M, Alberti S, Müller S (2020). The nuclear SUMO-targeted ubiquitin quality control network regulates the dynamics of cytoplasmic stress granules. Mol Cell.

[CR27] Kišonaitė M, Wild K, Lapouge K, Gesé GV, Kellner N, Hurt E, Sinning I (2023). Structural inventory of cotranslational protein folding by the eukaryotic RAC complex. Nat Struct Mol Biol.

[CR28] Kmiecik SW, Le Breton L, Mayer MP (2020). Feedback regulation of heat shock factor 1 (Hsf1) activity by Hsp70-mediated trimer unzipping and dissociation from DNA. EMBO J.

[CR29] Kolhe JA, Babu NL, Freeman BC (2023). The Hsp90 molecular chaperone governs client proteins by targeting intrinsically disordered regions. Mol Cell.

[CR30] Kolla S, Ye M, Mark KG, Rapé M (2022). Assembly and function of branched ubiquitin chains. Trends Biochem Sci.

[CR31] Kong K-YE, Fischer B, Meurer M, Kats I, Li Z, Rühle F, Barry JD, Kirrmaier D, Chevyreva V, San Luis B-J, Costanzo M, Huber W, Andrews BJ, Boone C, Knop M, Khmelinskii A (2021). Timer-based proteomic profiling of the ubiquitin-proteasome system reveals a substrate receptor of the GID ubiquitin ligase. Mol Cell.

[CR32] Koopman M, Janssen L, Nollen EAA (2021) Optogenetic manipulation of individual or whole population *Caenorhabditis elegans* worms with an under hundred-dollar tool: the OptoArm (preprint). Genetics. 10.1101/2021.03.19.435933

[CR33] Krakowczyk M, Lenkiewicz AM, Sitarz T, Marins Mussulini BH, Linke V, Malinska D, Szczepankiewicz A, Wydrych A, Nieznanska H, Serwa RA, Chacinska A, Bragoszewski P (2023) OMA1 protease eliminates arrested protein import intermediates upon depolarization of the inner mitochondrial membrane (preprint). Mol Biol. 10.1101/2023.06.08.543713

[CR34] Krzyzosiak A, Sigurdardottir A, Luh L, Carrara M, Das I, Schneider K, Bertolotti A (2018). Target-based discovery of an inhibitor of the regulatory phosphatase PPP1R15B. Cell.

[CR35] Kuiper EFE, Gallardo P, Bergsma T, Mari M, Kolbe Musskopf M, Kuipers J, Giepmans BNG, Steen A, Kampinga HH, Veenhoff LM, Bergink S (2022). The chaperone DNAJB6 surveils FG-nucleoporins and is required for interphase nuclear pore complex biogenesis. Nat Cell Biol.

[CR36] Labbadia J, Morimoto RI (2015). Repression of the heat shock response is a programmed event at the onset of reproduction. Mol Cell.

[CR37] Michaelis JB, Brunstein ME, Bozkurt S, Alves L, Wegner M, Kaulich M, Pohl C, Münch C (2022). Protein import motor complex reacts to mitochondrial misfolding by reducing protein import and activating mitophagy. Nat Commun.

[CR38] Morán Luengo T, Kityk R, Mayer MP, Rüdiger SGD (2018). Hsp90 breaks the deadlock of the Hsp70 chaperone system. Mol Cell.

[CR39] Morreale FE, Kleine S, Leodolter J, Junker S, Hoi DM, Ovchinnikov S, Okun A, Kley J, Kurzbauer R, Junk L, Guha S, Podlesainski D, Kazmaier U, Boehmelt G, Weinstabl H, Rumpel K, Schmiedel VM, Hartl M, Haselbach D, Meinhart A, Kaiser M, Clausen T (2022). BacPROTACs mediate targeted protein degradation in bacteria. Cell.

[CR40] Müller MBD, Kasturi P, Jayaraj GG, Hartl FU (2023). Mechanisms of readthrough mitigation reveal principles of GCN1-mediated translational quality control. Cell.

[CR41] Münch C (2018). The different axes of the mammalian mitochondrial unfolded protein response. BMC Biol.

[CR42] Patil PR, Burroughs AM, Misra M, Cerullo F, Dikic I, Aravind L, Joazeiro CAP (2023) Mechanism and evolutionary origins of alanine-tail C-degron recognition by E3 ligases Pirh2 and CRL2-KLHDC10. BioRxiv Prepr Serv Biol. 10.1101/2023.05.03.539038 10.1016/j.celrep.2023.113100 PMC10591846 37676773

[CR43] Rozales K, Younis A, Saida N, Meller A, Goldman H, Kellerman L, Heinrich R, Berlin S, Shalgi R (2022). Differential roles for DNAJ isoforms in HTT-polyQ and FUS aggregation modulation revealed by chaperone screens. Nat Commun.

[CR44] Sherpa D, Chrustowicz J, Qiao S, Langlois CR, Hehl LA, Gottemukkala KV, Hansen FM, Karayel O, von Gronau S, Prabu JR, Mann M, Alpi AF, Schulman BA (2021). GID E3 ligase supramolecular chelate assembly configures multipronged ubiquitin targeting of an oligomeric metabolic enzyme. Mol Cell.

[CR45] Sidrauski C, Acosta-Alvear D, Khoutorsky A, Vedantham P, Hearn BR, Li H, Gamache K, Gallagher CM, Ang KK-H, Wilson C, Okreglak V, Ashkenazi A, Hann B, Nader K, Arkin MR, Renslo AR, Sonenberg N, Walter P (2013) Pharmacological brake-release of mRNA translation enhances cognitive memory. eLife 2: e00498. 10.7554/eLife.00498 10.7554/eLife.00498 PMC3667625 23741617

[CR46] Sidrauski C, Tsai JC, Kampmann M, Hearn BR, Vedantham P, Jaishankar P, Sokabe M, Mendez AS, Newton BW, Tang EL, Verschueren E, Johnson JR, Krogan NJ, Fraser CS, Weissman JS, Renslo AR, Walter P (2015). Pharmacological dimerization and activation of the exchange factor eIF2B antagonizes the integrated stress response. eLife.

[CR47] Sitron CS, Brandman O (2019). CAT tails drive degradation of stalled polypeptides on and off the ribosome. Nat Struct Mol Biol.

[CR48] Sitron CS, Park JH, Giafaglione JM, Brandman O (2020). Aggregation of CAT tails blocks their degradation and causes proteotoxicity in S. cerevisiae. PLoS One.

[CR49] Sontag EM, Morales-Polanco F, Chen J-H, McDermott G, Dolan PT, Gestaut D, Le Gros MA, Larabell C, Frydman J (2023). Nuclear and cytoplasmic spatial protein quality control is coordinated by nuclear-vacuolar junctions and perinuclear ESCRT. Nat Cell Biol.

[CR50] Sui X, Prado MA, Paulo JA, Gygi SP, Finley D, Morimoto RI (2022) Global proteome metastability response in isogenic animals to missense mutations and polyglutamine expansions in aging (preprint). Biochemistry. 10.1101/2022.09.28.509812

[CR51] Sutandy FXR, Gößner I, Tascher G, Münch C (2023). A cytosolic surveillance mechanism activates the mitochondrial UPR. Nature.

[CR52] Taylor G, Cui H, Leodolter J, Giese C, Weber-Ban E (2023). ClpC2 protects mycobacteria against a natural antibiotic targeting ClpC1-dependent protein degradation. Commun Biol.

[CR53] Taylor G, Frommherz Y, Katikaridis P, Layer D, Sinning I, Carroni M, Weber-Ban E, Mogk A (2022). Antibacterial peptide cyclomarinA creates toxicity by deregulating the Mycobacterium tuberculosis ClpC1-ClpP1P2 protease. J Biol Chem.

[CR54] The Proteostasis Consortium, Overall coordination, Elsasser S, Elia LP, Morimoto RI, Powers ET, Harvard Medical School group (analysis), Finley D, University of California, San Francisco and Gladstone Institutes group I (chaperones, analysis), Mockler E, Lima L, Finkbeiner S, University of California, San Francisco group II (chaperones, analysis), Gestwicki JE, Northwestern University group (chaperones, analysis), Stoeger T, Cao K, The Scripps Research Institute group (chaperones, endoplasmic reticulum proteostasis, mitochondrial proteostasis, analysis), Garza D, Kelly JW, Stanford University group (chaperones, translation, mitochondrial proteostasis), Collier M, Rainbolt TK, Taguwa S, Chou CC, Aviner R, Barbosa N, Morales-Polanco F, Masto VB, Frydman J (2022) A comprehensive enumeration of the human proteostasis network. 1. Components of translation, protein folding, and organelle-specific systems (preprint). Bioinformatics. 10.1101/2022.08.30.505920

[CR55] The Proteostasis Consortium, Overall coordination, Elsasser S, Elia LP, Morimoto RI, Powers ET, Harvard Medical School group, Elsasser S, Finley D, University of California, San Francisco and Gladstone Institutes group I, Costa B, Budron M, Tokuno Z, Wang S, Iyer RG, Barth B, Mockler E, Elia LP, Finkbeiner S, University of California, San Francisco group II, Gestwicki JE, Northwestern University group, Richardson RAK, Stoeger T, Morimoto RI, The Scripps Research Institute group, Tan EP, Xiao Q, Cole CM, Massey LA, Garza D, Powers ET, Kelly JW, Stanford University group, Rainbolt TK, Chou CC, Masto VB, Frydman J, New York University group, Nixon RA (2023) A comprehensive enumeration of the human proteostasis network. 2. Components of the autophagy-lysosome pathway (preprint). Bioinformatics. 10.1101/2023.03.22.533675

[CR56] Thrun A, Garzia A, Kigoshi-Tansho Y, Patil PR, Umbaugh CS, Dallinger T, Liu J, Kreger S, Patrizi A, Cox GA, Tuschl T, Joazeiro CAP (2021). Convergence of mammalian RQC and C-end rule proteolytic pathways via alanine tailing. Mol Cell.

[CR57] Tsai JC, Miller-Vedam LE, Anand AA, Jaishankar P, Nguyen HC, Renslo AR, Frost A, Walter P (2018). Structure of the nucleotide exchange factor eIF2B reveals mechanism of memory-enhancing molecule. Science.

[CR58] Tsaytler P, Harding HP, Ron D, Bertolotti A (2011). Selective inhibition of a regulatory subunit of protein phosphatase 1 restores proteostasis. Science.

[CR59] Ulamec SM, Maya-Martinez R, Byrd EJ, Dewison KM, Xu Y, Willis LF, Sobott F, Heath GR, van Oosten Hawle P, Buchman VL, Radford SE, Brockwell DJ (2022). Single residue modulators of amyloid formation in the N-terminal P1-region of α-synuclein. Nat Commun.

[CR60] van den Boom J, Marini G, Meyer H, Saibil HR (2023). Structural basis of ubiquitin-independent PP1 complex disassembly by p97. EMBO J.

[CR61] Williams C, Dong KC, Arkinson C, Martin A (2023). The Ufd1 cofactor determines the linkage specificity of polyubiquitin chain engagement by the AAA+ ATPase Cdc48. Mol Cell.

[CR62] Wu Z, Berlemann LA, Bader V, Sehr D, Eilers E, Covallero A, Meschede J, Angersbach L, Showkat C, Michaelis JB, Münch C, Rieger B, Namgaladze D, Herrera MG, Fiesel FC, Springer W, Mendes M, Stepien J, Barkovits K et al (2022) LUBAC assembles a signaling platform at mitochondria for signal amplification and shuttling of NF-ĸB to the nucleus (preprint). Cell Biol. 10.1101/2022.05.27.493704

[CR63] Yagita Y, Zavodszky E, Peak-Chew S-Y, Hegde RS (2023). Mechanism of orphan subunit recognition during assembly quality control. Cell.

[CR64] Yuste-Checa P, Bracher A, Hartl FU (2022). The chaperone clusterin in neurodegeneration-friend or foe? BioEssays News Rev. Mol Cell Dev Biol.

[CR65] Yuste-Checa P, Trinkaus VA, Riera-Tur I, Imamoglu R, Schaller TF, Wang H, Dudanova I, Hipp MS, Bracher A, Hartl FU (2021). The extracellular chaperone clusterin enhances tau aggregate seeding in a cellular model. Nat Commun.

[CR66] Zavodszky E, Peak-Chew S-Y, Juszkiewicz S, Narvaez AJ, Hegde RS (2021). Identification of a quality-control factor that monitors failures during proteasome assembly. Science.

[CR67] Zhu PJ, Khatiwada S, Cui Y, Reineke LC, Dooling SW, Kim JJ, Li W, Walter P, Costa-Mattioli M (2019). Activation of the ISR mediates the behavioral and neurophysiological abnormalities in Down syndrome. Science.

